# Two Cases Illustrating the Production of Vowel and Consonant Sounds

**Published:** 1872-06

**Authors:** E. M. Moore

**Affiliations:** Rochester, N. Y.


### Miscellaneous.

### Two Cases illustrating the production of Vowel and Consonant sounds.

By Prof. E. M. MOORE, M. D., Rochester, N. Y.

The function of voice has always been a theme of interest to a wider circle of men than physicians. The most profound students of language, those who push their inquiries into the phonetic forms of the dim past, seeking to unravel the net of history in the twilight of the race, are perhaps more interested than any other class, except the pure physiologist. The students of Sanskrit have brought forward elaborate and excellent treatises upon phonation, written more than three thousand years ago, pushing the inquiry as far and as accurately as mere observation could go. Since the discovery of the laryngoscope the whole subject has undergone and is now undergoing a complete revision by the practice of auto-laryngoscopy, and we shall undoubtedly have the whole topic vastly advanced. Indeed it may be so far, as to enable us successfully to initiate an inquiry into an anatomical explanation of certain phonetic laws, which some profound German scholars have propounded, and also lead to the discovery of others. These men look to the physiologists, in hopes that a law of anatomical and physiological relation to sounds may be discovered, upon which to rest a solid foundation for the expansion of ethnology and comparative philology.

The unanimity of opinion among physiologists, who have given their attention to the function of the larynx in the production of the voice, has led me to note two cases of striking interest, and so conclusive in their character, that I think they will be regarded as an offset to the experiments and observations that have heretofore been made, and upon which opinion has rested.—They have the merit of substituting fact for inference, and, indeed, fact bearing the potency of an experimentum crucis. In order, however, to the full understanding of the question, I will take Carpenter as the authority for the standard opinions, and recite his view on the subject at the point I propose to elucidate.

The doctrine of the reed-sound in the production of the laryngeal voice, it seems to me, is firmly settled and universally admitted; the proof resting not only upon the form of the organ, but on the production of sound from the artificial larynx. Here at the vocal chords, the body of sound known as the sonant voice, in contradistinction to the whisper, is undoubtedly made. But the especial point to which this paper is directed is not this, but to the correction of opinion with reference to the production of vowel-sounds. These are asseverated to result from the passage of air along the mouth, and to be the effect of its modification, including, or course, the pharynx and nose as factors. In proof of this I quote Carpenter.

"The vowel sounds are continuous tones modified by the form of the aperture through which they pass out; while in sounding consonants the breath suffers a more or less complete interruption in its passage through parts anterior to the larynx. Hence the really simple vowel-sounds are capable of prolongation during any time that breath can sustain them. This is not the case, however, with the real diphthongal sounds (of which it will presently appear that the English *i* is one), whilst it is true of some consonants. It seems to have been forgotten by many who have written on this subject, that the laryngeal voice is not essential to the formation of either vowels or consonants; for all may be sounded in a whisper. It is evident, therefore, that the larynx is not primarily concerned in their production, and this has been fully established."

Again he says: "That the vowel-sounds are produced by simple modifications in the form of the external passages is easily proved, both by observation and imitative experiment. When the mouth is opened wide, the tongue depressed, and the velum palati elevated, so as to give the freest possible exit to the voice, the vowel *a* in its broadest form *(ah)* is sounded. On the other hand, if the oral aperture be contracted, the tongue being still depressed, the sound *oo*, the continental *u*, is produced. If attention be paid to the state of the buccal cavity during the pronunciation of the different vowel-sounds, it will be found to undergo a great variety of modifications, arising from varieties of position of the tongue, the cheeks, the lips, and the velum palati. The position of the tongue is, indeed, one of the primary conditions of the variation of the sounds, for it may be easily ascertained that, by peculiar inflections of this organ, a great diversity of vowel-sounds may be produced; the other parts remaining the same. Still there is a certain position of all the parts which is most favorable to the formation of each of these sounds; but this could not be expressed without a lengthened description. The following table, slightly altered from that of Kempelen, expresses the relative dimensions of the *buccal cavity*

and of the *oral orifice*, for some of the principal of these—the number 5 expressing the largest size, and the others in like proportion:—

| Vowel-sounds. | Oral opening. | Size of Buccal cavity. |
|---|---|---|
| *a* as in ah | 5 | 5 |
| *a* " name | 4 | 2 |
| *e* " theme | 3 | 1 |
| *o* " cold | 2 | 4 |
| *oo* " cool | 1 | 5 |

"These are the sounds of the five vowels, *a*, *e*, *i*, *o*, *u*, in most continental languages; and it cannot but be admitted that the arrangement is a much more natural one than that of our own vowel-series." Having made this quotation, I proceed at once to give the first case, bearing directly on this point:—

Edward Larkin, æt. 56, born in Ireland, April 14, 1871, attempted suicide with a razor. A cut was made immediately above the thyroid cartilage, extending an inch each side of the median line horizontally, and shaving off the epiglottis at its base. The muscles below and above, by their tonic power, soon made an oval opening, two inches by ¾ of an inch. Swallowing became impossible. On the fourth day he was brought to St. Mary's Hospital, having taken no food. No attempt was made to bring the edges together, trusting to the granulating process. A tube, introduced through the opening into the œsophagus, conveyed food to the stomach. This process was continued for about five weeks, when swallowing became possible, although the opening, which had become nearly circular, was about half an inch in diameter. At the present time (June 10, 1871) he swallows solid food, but part of the liquid food will run out of the opening. At any time he was able to talk by bending his head forward and temporarily closing the opening. When the head was thrown back, he immediately lost this power.

The experiment of enunciation of the alphabetical sounds, with the head thrown back, was made with the vowels first. The four sounds of *a* as in *all*, *arm*, *age*, *at*, were at once distinctly rendered. *E*, next attempted, always resulted in *a* as in *age*. [This, I think, must be put to the account of the Hybernian larynx.]

*I* is a diphthong, and was clearly rendered.

*O*, both long and short, was well brought out as in *note* and *not*. The short *u* as in *cut*, and represented by nearly all the other vowels, was also well pronounced.

*Y* as *I*.

The diphthong were more difficult, and some could not be uttered; *ou* as in *our* was pretty well pronounced; *oo*, pure vowel, was uniformly rendered as *o* in *note*.

*Oi*, as in *noise*, could scarcely be distinguished.

The alphabet, taken in order, resulted in the enunciation of the vowel element. Thus our letter *B* was pronounced *a*, as also *C* and *D*. There was absolutely no consonant uttered. The breathing, which is represented by the letter *H*, was perfectly rendered. It was clear and smooth, and when joined to a vowel was spoken loudly and clearly. Thus, *Ha* and other sounds of *a*, *Hi* and *Ho*, also *Hu short*; were pronounced with ease. *K* and *G* are considered guttural aspirates. Any attempt to produce them resulted in the pure aspirate.

These experiments were repeated on two different occasions, a few days apart, and with the same results. But on the second trial it was suggested that some air might pass into the mouth, and a curtain of buckskin was fittet to the upper part of the aperture, thus cutting off all the air that might reach the mouth. The result was the same.

As regards the finish of the tones called vowels, there was clearly a defect. This might be due to the thickening of the mucous membrance, incident to the inflammation in its immediate neighborhood—a good and quite sufficient cause. Or it might be explained by the absence of the modification by the mouth, tongue, and other parts, which have been supposed to create the sounds, but which, as is here shown, can only polish and complete what has been begun. But a little practice will enable one to hold the parts above the larynx perfectly quiet, and still produce the vowel-sound. This case is so pathognomonic, if I can apply such a term, that it must reverse and settle opinion upon the seat of certain vowel-sounds.

The number of sounds that Larkin was able to enunciate is limited; but I will now cite another case that, from its peculiar character, supplements the one just detailed, and by its results confirms the belief that Larkin's ability to enunciate as described would exist in all others, thus placing the seat of such vocalization as normal in all.

During the year 1856 I was called into Orleans Co. to see Edward Matthews, who was suffering from urgent dyspnœa. I arrived at his house in the night, and at once opened the crico-thyroid space. The relief was sufficient, and I did not see him again for a long time. Two years after, as I am informed, a piece of bone was removed from the opening, which had been kept free by a tube. This seemed to be lodged in the larynx somewhere, but the statement is too indifinite to be thoroughly understood. Nevertheless, there was a great abatement of unpleasant symptoms from soreness and dyspnœa. But slowly and gradually the glottis became closed, and for more than ten years a cicatrix has cut off the air from the trachea into the mouth, the closure being at the base of the larynx and absolute, not the least air passing through. Mr. Matthews, who is now forty-four years of age, is a man of intelligence, and has made great and constant efforts to enunciate clearly, and has improved much of latter years. Of course, every sound is a whisper, and also short, for the air is driven by the buccinators and the muscles of the tongue and the supply of air is small. From this man we would expect to hear the consonants, if he could speak at all, and this is essentially true.

*R* and *L* he cannot pronounce, as they require a continuous tone, and are interested in a quiescent state of the buccinators, which are the chief means of forcing the air out. But even these sounds can be faintly traced in combination with others, in the construction of a word. The pure breathing represented by the letter *H* cannot be made at all, or in any combination. The single vowel-sounds of *a*, as in *arm*, *at*, *all*, and *age*, cannot be rendered at all. A blowing sound was the result of the effort, and when the finger was placed over the aperture in the trachea there was absolute silence. The celebral command was obviously to the larynx. *O* was also silent, or resulting in *oo*, as in *ooze*. *I*, as in *isle*, was also silent when the aperture was closed, or a mere blowing sound if opened. *E* clearly enunciated, and this vowel is made in the front part of the mouth, by throwing the tip of the tongue against the lower incisors and curling it upwards. While fixed, the continuous tone *E* results.

The nameless vowel represented by *u* as in *nut*, *e* as in *err*, *i* as in *sir*, and *o* as in *honor*, sometimes styled the urvocal, could not be uttered except in connection with consonants.

Short *o* as in *not*.

" *e* " *net*.

" *i* " *nit*, could be rendered in conjunction with consonants, but, with the exception of short *e*, could not be rendered individually. The diphthongs were much more successfully pronounced than by Larkin.

Thus the soft *u* as in *gratitude* is a diphthong composed of *e u* (*oo*) and was rendered beautifully.

*Oo* is really a very pure vowel, perhaps the most of any, if we except *e*, but it is not so placed in our catalogues, and I can hardly understand why, unless it happens to be represented by a double character. This is made at the lips exclusively, as in *ooze*, and was rendered with great perfection.

*Oi* as in *noise*, and *ou* as in *our*, both failed.

As might be expected, the consonants are generally pronounced, most of them individually, but some only in connection with vowels.

B, C, D, F, G, K, P, T, X, Z, and V, pronounced as words and pure. But H not all. L. and R. very faint, when combined with *e* long.

M and N in connection with vowels only.

The consonants, P and T, made in the front part of the mouth, were more easily managed than the others. Accordingly the vowels within his capacity were sure to be brought out best between them. Thus:—

Pet....................................................Pretty well.
Pit.......................................................“ “
Pot.......................................................“ “
Put.......................................................“ “
Peet....................................................Admirably.
Pout....................................................Not at all.
Peut....................................................Beautifully.
Pat as *Put* or *Pot.*
Pate, not at all, or as *Peet.*
Th, as in Thing and With..............................Well.
Ch, as in Church, and Sh, as in Shall..............Well.
Nt, as in *Sent*.............................................Well.

The conclusions that I arrive at from these two striking cases are these:—

1st. That the larynx is not only the generator of voice (so called), but the actual seat of vocalization for the vowels *a*, in all its forms, *i* long, *o* long, and the pure aspirate. Also the short vowels, which are also explosive, as *i* is in *sit*, *o* in *not*, and nameless one or urvocal.

2d. That these sounds receive a finish in the pharynx, nose, and mouth; with the exception of the pure breathing and the short vowels.

3d. The consonants are all made above the larynx.

4th. The vowel *e* long is made purely in the front part of the mouth, also the vowel *oo* and the diphthong *eu*, which is composed of these two elements,

5th. The short vowels (*e* in *met*) (*i* in *sit*) (*u* in *nut*) (*o* in *not*) can be made in the front part of the mouth.

6th. The urvocal can probably be made in several places from the larynx to the front of the mouth.

These propositions are nearly all mere statements of facts, but some are inferences.

The observation on Edward Larkin were made in the presence of my colleagues Drs. Casey and Carroll of St. Mary's Hospital, and also of Prof. Lattimore of the Rochester University. They were repeated on two occasions, a few days apart, but became after a short time impossible from closure.

Those on Mr. Matthews have been repeated on three occasions, within the last three months: on one in the presence of my colleagues in the Buffalo Medical College and several physicians and students of medicine, and on another, in the presence of Prof. Mixer of the Rochester University, and Prof. Palmer of the Normal School at Brockport—two gentlemen whose interest in the case arose from the fact, that they were the teachers of languages in their respective institutions. Doubtful sounds have been carfully excluded, and I feel sure that no observations have been retained which are not clear and decided beyond cavil. *The Medical Record.*

